# Dramatically Enhanced Superconductivity in Elemental Bismuth from Excitonic Fluctuation Exchange

**DOI:** 10.1038/s41598-017-11269-y

**Published:** 2017-09-08

**Authors:** S. Koley, M. S. Laad, A. Taraphder

**Affiliations:** 1 Instt. Math. Sciences Taramani, and Hoormi Bhabha National Institute, Chennai, 600113 India; 2 Department of Physics and Centre for Theoretical Studies, Indian Institute of Technology, Kharagpur, 721302 India; 3 Department of Physics, St. Anthony’s College, Shillong, Meghalaya 793001 India

## Abstract

Motivated by the remarkable discovery of superconductivity in elemental Bismuth at ambient pressure, we study its normal state in detail using a combination of tight-binding (TB) band-structure supplemented by dynamical mean-field theory (DMFT). We show that a two-fluid model composed of preformed and dynamically fluctuating excitons coupled to a tiny number of carriers provides a unified rationalization of a range of ill-understood normal state spectral and transport data. Based on these, we propose that resonant scattering involving a very low density of renormalized carriers and the excitonic liquid drives logarithmic enhancement of vertex corrections, boosting superconductivity in *Bi*. A confirmatory test for our proposal would be the experimental verification of an excitonic semiconductor with electronic nematicity as a ‘competing order’ on inducing a semi-metal-to semiconductor transition in *Bi* by an external perturbation like pressure

## Introduction

Rhombohedral Bismuth (Bi) has recently acquired prominence in a variety of contexts. Like graphite, elemental Bi also shows a magnetic field- as well as a pressure-induced metal-insulator-like transition^1^ with a large magneto-resistance, and electron fractionalization in high magnetic fields^2^. The remarkable discovery of superconductivity (SC) at ambient pressure in Bi at very low temperature (*T*_*c*_ below 0.53 mK)^3^, in a lowest carrier-density elemental system to date, adds to the range of novel behaviors exhibited by this ‘simple’ system. The following experimental features of this discovery: (*i*) *T*_*c*_ ≃ 0.5 mK, while a naive BCS estimate^3^ would yield ${T}_{c}^{BCS}\simeq 100$ nano-K, and (*ii*) *B*_*c*_(0)/*T*_*c*_ is experimentally found to be 9.4 T/K, greatly enhanced over the BCS estimate of 0.79 T/K, strongly point to the possibility of SC in Bi *not* being of the standard BCS variety and, in fact, it would be more aptly characterized as being in a non-adiabatic limit of pairing theory if electron-boson (*e*.*g*, phonon) coupling were to be invoked as the dominant pairing glue.

The unique electronic properties of *Bi* arise from successive distortions of a higher-symmetry simple-cubic structure, which, except for a tiny change in the *b/d*-parameter^4^ (here, *d* is the spacing of the (111)-rhombohedral planes of one sublattice, while *b* is the displacement of the other sublattice (111)-planes from these) remains unchanged from high (300 K) to low (4.2 K) temperature. This is a small relative displacement along the body diagonal, which doubles the unit cell. By itself, this would drive *Bi* into a Peierls-like band semiconductor. But additional rhombohedral shear (which exists from *T* = 300 K down to 4.2 K) causes further lowering of symmetry, allowing valence and conduction band overlap^1^ in good accord with band structure studies^5, 6^, and explains why *Bi* is metallic. Due to extremely small Fermi pockets (of size 10^−5^ of the Brillouin zone) and tiny carrier density (3 × 10^17^ cm^−3^), the long mean-free path accounts for its small low-*T* resistivity. One might then think that traditional one-electron band structure is an adequate framework to understand its electronic properties.

However, careful perusal of extant data points toward a much more interesting situation. Early data^7^ show that the electrical resistivity ${\rho }_{dc}(T)\simeq {\rho }_{0}+A{T}^{2}$ for *T* < 50 K. But very unusual behavior at low *T*, with ${\rho }_{dc}(T)\simeq {T}^{5}$ was recently seen: within an itinerant picture involving long-range coulomb interaction, an attempt was made to rationalize this behavior in a plasmaron picture^8^, where a *plasmaron* is considered to be a collective excitation involving strong coupling between the charge carriers and plasmons^8^. Optical data raise additional issues in this context^9^. While a tiny Drude component is visible at low energy, finding of sizable mid-infra-red (mid-IR) absorption is inexplicable in a free-electron framework. Given the tiny Fermi energy $\simeq 25$ meV, a large *T*-dependent transfer of spectral weight over a much larger scale $\simeq 300$ meV also presents a challenge for the standard (uncorrelated band) view.

On the theoretical front, first-principles density-functional theory (DFT) and Slater-Koster tight-binding fits^6^ conclusively show that: (*i*) there are multiple tiny pockets in *Bi*, with two being almost perfectly compensated, and the others supplying a tiny additional number of carriers. Moreover, the importance of spin-orbit coupling (SOC) is shown by the fact that the correct positions of the e(h) pockets are only found when sizable SOC, *O*(1.3) eV, is included in band calculations. Thus, DFT + SOC calculations indeed yield good description of *ground state* properties. In particular, they yield the correct shape and size of the carrier pockets at the *T* and *L* points in the Brillouin zone. However, it is also well known that such a ground state theory can fall short of describing the dynamical excitation spectrum and, consequently, the finite-temperature (*T*) responses^10^. This is especially true when coulomb interaction-induced bound state formation is involved: in the specific context of *Bi*, it is long known^9^ that the dominant interaction between conduction and valence band carriers in *Bi* is of *short-range excitonic* character. This raises the possibility of exciton bound state formation, but its role in *Bi* has never been satisfactorily addressed. While DFT calculations work well in moderately correlated solids, they are, by construction, inadequate when such (*e*.*g*, excitonic) bound states appear due to correlations. Generically, one also expects a symmetry-adapted coupling of such interband excitons to intervalley phonons^11^ in *Bi*: since the ratio of the phonon Debye frequency *k*Θ_*D*_ to *E*_*F*_ is large ($\simeq 0.5$ in *Bi*), sizable self-energy effects due to this additional coupling will also influence normal state responses above *T*_*c*_. As far as SC is concerned, the huge enhancement of the SC transition temperature, ${T}_{c}^{exp}/{T}_{c}^{BCS}\simeq {10}^{3}\mbox{--}{10}^{4}$, and the large discrepancy^3^ between the measured and calculated (within BCS theory) ratio of the upper critical field to *T*_*c*_ also mandates a non-adiabatic ‘strong coupling’ SC beyond a traditional BCS view.

These limited observations must constrain theoretical modelling: both unconventional metallicity and SC must find explication in a picture based on (*i*) the special band structure of Bi and (*ii*) strong scattering (electron-hole and/or electron-phonon interaction) processes beyond DFT and, in particular, microscopic processes which generate a ${\rho }_{dc}\simeq {T}^{5}$ should also be involved in generating the SC pair glue.

Our first observation is that two of the multiple electron(hole) pockets of Bi are almost perfectly compensated, leading to a situation famously encountered in transition-metal dichalcogenides (TMD), where preformed excitonic liquid (PEL) driven charge-density-wave (CDW) states are ubiquitous^12–14^. Band calculations reveal a total of four pockets in *Bi*, of which two (electron and hole) exhibit a propensity for an excitonic instability. Were there only these two pockets, one would have expected an electron-hole attraction-mediated excitonic insulator. However, the remaining pockets now supply a tiny number of additional carriers, leading to a physical picture of *an incipient excitonic insulator self-doped with a tiny number of carriers*. Thus, we are led to a model of a self-doped excitonic insulator (EI) in the intermediate coupling limit, where novel physics can arise from strong scattering between carriers and preformed but uncondensed excitons. In this report, we establish this using tight-binding-plus dynamical mean-field theory (TB-DMFT) calculations as done earlier^12–14^. Armed with very good accord with normal state transport, we elucidate a ‘strong coupling’ electronic mechanism, wherein strong resonant scattering between carriers and preformed excitons enhances vertex corrections, boosting the SC *T*_*c*_ in *Bi*.

**Figure 1:**
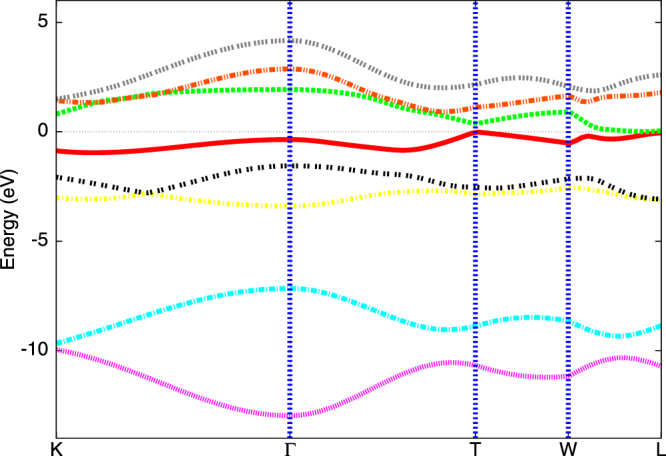
Tight binding (TB) bands of *Bi* in the rhombohedral structure including spin-orbit coupling (SOC) as done in ref. 6. For the effective two-band model incorporating excitonic correlations within DMFT calculations, we use only the two (red and green) bands crossing the Fermi energy, *E*_*F*_(=0).

We begin with a tight-binding description of the DFT band structure^6^ by using the Slater-Koster (SK) fit with form factors and parameters *including* SOC as in earlier work (given in SI Table I ^6^). The resulting band structure in Fig. 1 excellently reproduces all the pockets seen in full DFT calculations, constituting the appropriate band structural input for the correlation calculations. Guided by the discussion above, we focus on inter-band excitonic correlations. The two-band Hubbard model incorporating the two bands crossing the Fermi energy is *H* = *H*_0_ + *H*_1_, with

1 $${H}_{0}=\sum _{(\mu ,\nu =\mathrm{1,2),}k,\sigma }({\varepsilon }_{\mu }(k){c}_{k\mu \sigma }^{\dagger }{c}_{k\nu \sigma }+h\mathrm{.}c)+{\rm{\Delta }}\sum _{i}({n}_{i\mathrm{,1}}-{n}_{i\mathrm{,2}})$$

where *ε*_1_(*k*) = *E*_*p*_ + 3*V*_*ppσ*_cos $(\frac{\sqrt{3}{k}_{x}}{2})$ cos $(\frac{{k}_{y}}{2})+{V}_{pp\pi }$ $[\cos (\frac{\sqrt{3}{k}_{x}}{2})\cos (\frac{{k}_{y}}{2})+2\,\cos \,{k}_{y}]$ (represented by the green line in Fig. 1), *ε*_2_(*k*) = *E*_*p*_ + *V*_*ppσ*_ $[\cos (\frac{\sqrt{3}{k}_{x}}{2})\cos (\frac{{k}_{y}}{2})+2\,\cos \,{k}_{y}]$ + 3*V*_* ppπ*_cos $(\frac{\sqrt{3}{k}_{x}}{2})$ cos $(\frac{{k}_{y}}{2})$ (red line in Fig. 1) where E _*p*_ is on-site energy (−9.643 eV), V_*ppπ*_ and V_*ppσ*_ (2.271 eV and −0.679 eV, respectively) are third nearest neighbour hoppings and the interband matrix element ${V}_{12}(k)\,\backsimeq \,2i\sqrt{3}\,\sin (\frac{\sqrt{3}{k}_{x}}{2})\sin (\frac{{k}_{y}}{2})$, as extracted earlier^6^ from an SK fit. Here *c*_*k*_ and ${c}_{k}^{\dagger }$ represent anihilation and creation operator and *μ*, *ν* represent the band indices for the two bands crossing *E*_*F*_(=0) in Fig. 1. The interaction terms are

2 $${H}_{1}=\sum _{\mu =1,2}{U}_{\mu ,\mu }\sum _{i}{n}_{i,\mu ,\uparrow }{n}_{i,\mu ,\downarrow }+U^{\prime} \sum _{i}{n}_{i,1}{n}_{i,2}$$

taken to be dominantly local with the understanding^9^ that the coupling between valence and conduction band carriers is of short-range excitonic character, as pointed out above. Here *U*_1,1_ = *U*_2,2_ and *U*′ are the intra- and inter-band Coulomb interactions, *n*_*i*,*μ*_ = ∑_*σ*_ *n*_*i*,*μ*,*σ*_.

It is also long known that coupling of carriers to intervalley phonons is significant in *Bi* ^11^. Since the dimensionless measure of the electron-phonon (e-p) coupling, defined as $g={\omega }_{D}/{E}_{F}\simeq 0.5$ (*ω*_*D*_ the Debye frequency, and *E*_*F*_ = Fermi energy) is actually large in Bi^3^, one is in a non-adiabatic limit of the electron-phonon problem.This intermediate coupling regime is also interesting in that in contrast to the anti-adiabatic (*g* → ∞) or the adiabatic (*g* → 0) limits, one cannot “integrate out” these phonons to give a further e-h attraction *O*(*g* ^2^/*ħω*_*D*_). One must solve *H* including an explicit *e*-*p* term by coupling the intervalley phonons to the *k*-dependent hybridization as done in earlier work^13, 14^. Technically, coupling of inter-band excitons to phonons is described by adding a term ${H}_{e \mbox{-} p}=g{\sum }_{i,\sigma }{c}_{i,1,\sigma }^{\dagger }{c}_{i,2,\sigma }({A}_{i}+{A}_{i}^{\dagger })$ to *H* above, where ${A}_{i},{A}_{i}^{\dagger }$ are the intervalley phonon operators. We have followed our earlier procedure^13, 14^ to incorporate the sizable e-p coupling effects for *Bi* in the two-band Hubbard-like model above within the DMFT procedure (see SI(A) for details).

## TB+DMFT Results and Transport

We solve the two-band model above using DMFT, with multi-orbital iterated perturbation theory (IPT) as the ‘impurity’ solver. Though not numerically exact, it works very well in real multi-band cases for all temperatures and band-fillings, especially in cases where there is a sizable crystal-field splitting between the bands (here, between valence and conduction band). The bands that serve as a band-structural input to the correlation calculations are obtained by diagonalizing the one-electron part of the two-band model above, and labelled henceforth as “*a*” and “*b*” band states. IPT is a fast solver and its efficacy in a wide range of real systems is known^13–15^. In contrast, though state-of-the-art continuous-time quantum Monte Carlo (CTQMC) solvers are much more “numerically exact”, they cannot access temperatures below *O*(20) K at present^16^, which makes them unsuitable for investigation of the low *T*(<1 K) behavior in *Bi*. We choose *U*_1,1_ = *U*_2,2_ = 0.5–0.7 eV (henceforth we use the notation *U* = *U*_1,1_ = *U*_2,2_) and *U*′ = 0.1–0.2 eV as appropriate parameters, and while ab-initio estimates will yield more precise estimates, our present choice is physically motivated. Given widths of *O*(1.0–1.5) eV for the valence (VB) and conduction (CB) bands, we are in the intermediate coupling limit of the two-band model. This is precisely the case where DMFT works best (for specific studies in the BCS-BEC crossover, see ref. 17).

**Figure 2:**
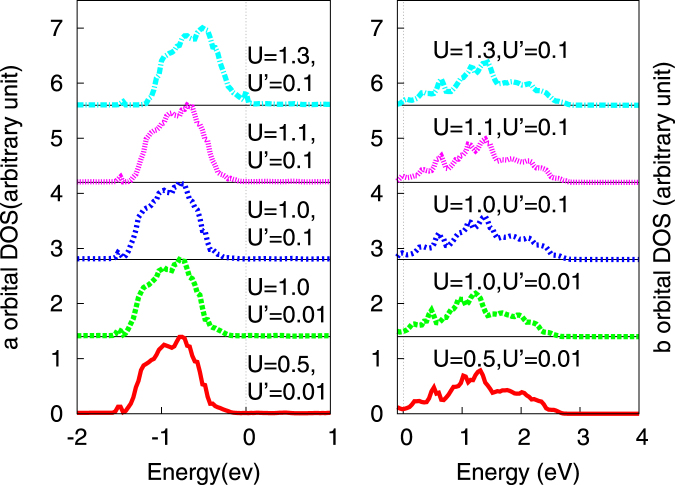
The orbitally resolved density of states from TB+DMFT at various U and U′.

In Fig. 2, we show the TB+DMFT local density-of-states (LDOS) as *U*, *U*′ are cranked up. While correlations gradually close the band gap for the VB (‘*a*’-orbital) as expected^18^, they reduce the LDOS at *E*_*F*_ for the CB (‘*b*’ orbital): these contrasting behaviors are direct consequences of distinct effects of local electronic correlations on band-insulating and metallic subsets of the non-interacting band structure. Around $U\simeq {W}_{b}$, the *b*-fermion band-width, we observe eventual opening of a *Mott*-like gap in the LDOS, seen by the fact that Im Σ_*b*_(*ω*) develops a pole structure at *E*_*F*_ in this case. Correspondingly, the *a*-fermion states retain metallic character: this would be an interesting manifestation of orbital-selective Mott physics in semi-metals and, were such a strong coupling regime to be realized, would open up the possibility to development of novel instabilities with concomitant competing orders^19, 20^ as, for instance, in Fe-arsenides. However, this is *not* the regime applicable to *Bi*, and so we concentrate on the smaller *U* regime.

**Figure 3:**
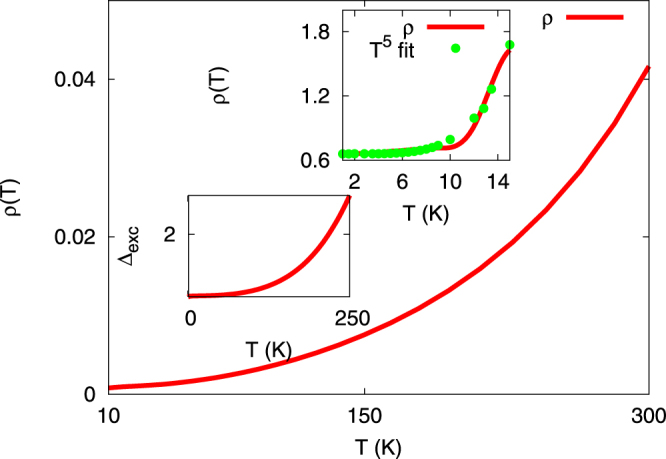
*DC* Resistivity for *Bi* within TB+DMFT: a *T*-dependent crossover from ${\rho }_{dc}(T)\simeq T$ at ‘high’-*T* to a correlated Landau-Fermi liquid like ${\rho }_{dc}(T)\simeq {T}^{2}$ below $T\simeq 90$ K is followed by ${\rho }_{dc}(T)\simeq {T}^{5}$ form at very low *T* (see inset for comparison with data points) is clear. The lower-left inset shows the *T*-dependent *local* interband excitonic average: onset of ${\rho }_{dc}(T)\simeq {T}^{5}$ correlates with a decreasing excitonic average. The y-axis of both the insets are divided by 10^−3^.

Choosing *U* = 0.5 eV, *U*′ = 0.15 eV, we next compute the *dc* resistivity using the Kubo formalism in DMFT. This task is facilitated by the finding^21^ that irreducible vertex corrections apprearing in the Bethe-Salpeter equations for conductivities are negligible and can be ignored to a very good approximation. Interestingly, as shown in Fig. 3, we find that a linear-in-*T* behavior of *ρ*_*dc*_(*T*) at ‘high’ *T* ≥ 40–50 K smoothly crosses over to a Fermi-liquid-like *T* ^2^ behavior up to about 10 K and, remarkably, exhibits a further low-*T* crossover to a ‘good’ metal with ${\rho }_{dc}(T)\simeq {T}^{5}$. This is in very good accord with experimental trends, and mandates deeper microscopic rationalization. To cement the link between transport and excitonic liquid fluctuations, Fig. 3 shows the *excitonic* average, computed as Δ_*exc*_ = (−1/*π*)∫*dω* Im *G*_12_(*ω*) (*G*_12_(*ω*) is itself computed from two-band DMFT(IPT) as previously done for TMDs^13, 14^). We find a clear correlation between Δ_*exc*_(*T*) and the *T*-dependence of *ρ*_*dc*_(*T* ) over a wide *T* range. This provides strong theoretical evidence linking the *dc* resistivity to microscopic processes involving scattering of the tiny number of carriers off well-formed and quasi-local excitonic correlations: at high-*T*, the latter are incoherent, leading to a quasi-linear-in-*T* resistivity, while increasing one-fermion coherence via suppression of incoherent excitonic fluctuations provides a rationalization for onset of enhanced metallic coherence, and the near *T* ^2^ behavior at intermediate *T*.

Remarkably, however, we also obtain the ${\rho }_{dc}(T)\simeq {T}^{5}$ dependence at very low *T*, the latter correlated with a reduced excitonic fluctuation at low *T*. Thus, we identify a new element: the *T*-dependence of sizable and dynamical ‘preformed’ excitonic correlations governs the *T*-dependent resistivity in a wide *T* window. Our proposal is distinct from: (*i*) the plasmaron view^8^, where weak-coupling RPA-like analysis is employed to get ${\rho }_{dc}\simeq {T}^{5}$ from long-range interactions, and (*ii*) pure e-p coupling models, which could, in principle, also yield similar behavior when $T\ll {{\rm{\Theta }}}_{D}$, something that may also obtain in *Bi*. We emphasize that e-p coupling *is* included on an equal footing with local excitonic correlations in our approach. We propose that a way to distinguish between these distinct scenarios could be *T*-dependent tunnelling measurements at small-to-intermediate *T*: as a function of *T*, the conductance *g*(*V*) = *dI*/*dV* would, in a picture involving coupling of carriers to any bosonic mode(s), show finite-voltage (energy) peak-dip-hump features. The energies and spectral weights of such features could be compared with estimates from different bosonic channels, allowing a determination of the most important fermion-boson scattering channel. However, since there will always be a symmetry-dictated coupling of interband excitons to intervalley phonons, one also generally expects *two* bosonic modes at different energies to show up in *g*(*V*): their relative weights provide an estimate of the relative importance of carrier-exciton vis-a-vis carrier-phonon coupling.

**Figure 4:**
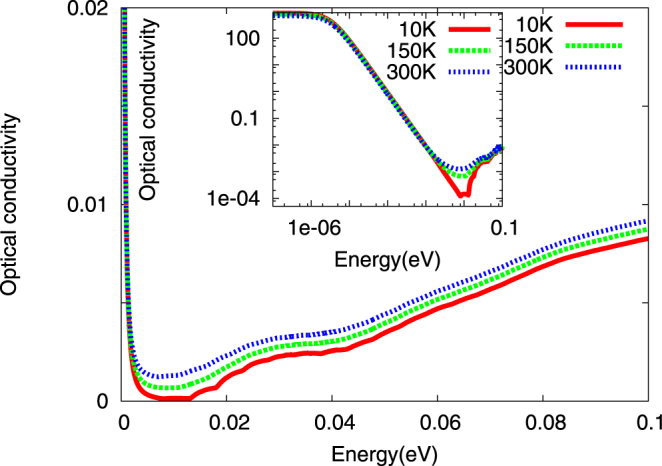
TB+DMFT Optical Conductivity in the normal state for *Bi*. A tiny Drude-like contribution is followed by inter-band absorption: this feature onsets at ≃15 meV and originates from strong resonant scattering between the tiny number of carriers and interband excitons in DMFT. The pre-peak in the absorption, centered at ≃10 meV, also originates from the same mechanism. The inset reveals the same features in more detail on a log-log plot. These findings are in very good accord with data^9^.

Further support for our view arises when we compare the DMFT optical conductivity, *σ*_*xx*_(*ω*) in Fig. 4 as a function of *T* with published data^9^. Since we have kept only the two lowest bands crossing *E*_*F*_ from the SK fit, we do not expect accord at higher energies, but can readily make a comparison for the relevant energy range (few hundred milli-eV) of interest. Specifically, up to about 100 meV, our result matches quite well with data, including (*i*) the plasmon edge, (*ii*) the detailed optical lineshape as a function of energy up to about 100 meV, (*iii*) sizable optical spectral weight transfer upon raising *T*. The plasmon-like features are clearly visible on both regular and log-log plots as a clear absorption onset at ≃15 meV at low *T* = 10 K. Interestingly, it is also *preceded* by a ‘prepeak’ structure, centered at ≃10 meV, in good accord with observations^9^. This prepeak feature is also washed out with increasing *T*, in accord with data. Clear spectral weight transfer up to ≃200 meV is also found: given the tiny *E*_*F*_ in *Bi*, this is quite a large energy scale, attesting to considerable dynamical correlations. Finally, we find an isosbestic point around 5.0 meV as a function of *T*, which is another characteristic signature of dynamical electronic correlations that could be tested in extant work.

Taken together, our results strongly support the idea that a common underlying scattering process involving tiny number of carriers and uncondensed fluctuating interband excitons may be operative in *Bi*. That the dominant interaction in *Bi* has a short-range excitonic character is well known^9^. Our results show that it seems to be a sufficient *minimal* input to understand transport in *Bi*, and ties in our view with plasmarons^8^. Generally, in a correlated metal, appearance of mid-infra-red features (*e*.*g*, DMFT for one-band Hubbard type models) involve interband transitions between low-energy itinerant and high-energy localized Hubbard-band states^10^. In our case, they arise from transitions involving carrier states scattering off the incoherent and interband excitons involving precisely the renormalized VB and CB in the *two-band* Hubbard-like model, since *σ*_*xx*_(*ω*) in DMFT is just a direct convolution of the one-particle DMFT spectral functions. In *Bi*, this feature occurs at a very low energy because of the tiny *E*_*F*_. The interband electronic excitations involving carriers coupled to such exciton-like entities have been christened *plasmaron* in prior work. We find that this characterization is not in conflict with our excitonic fluctuation picture, since we have now shown that it can be well described by analyzing effects of predominantly local and sizable excitonic liquid-like correlations in a quasi-realistic model for *Bi*. Finally, since excitonic correlations can trigger changes in lattice parameters, whether development of excitonic (liquid) correlations can rationalize the small but unexplained dependence of the rhombohedral parameter (*u*)^4^ on *T* is interesting to inquire, but out of scope of the present work.

Buoyed by the agreement with the phenomenology so far, we venture to propose a specific model for superconductivity in *Bi*. To do so, we need to generate an effective pairing interaction arising from excitonic and/or phonon fluctuation exchange. Given the efficacy of the excitonic liquid view above for the normal state, and that the exciton formation energy scale (${{\rm{\Omega }}}_{ex}\simeq {{\rm{\Delta }}}_{exc}$) is quite large, we are clearly in the non-adiabatic limit. This precludes a BCS-like instability to SC in *Bi*. In the normal state, the interband excitons involving the VB and CB form an isospin *T* = 1/2 degree of freedom, described by ${T}^{+}={\sum }_{k,\sigma }{a}_{k\sigma }^{\dagger }{b}_{k\sigma },{T}^{-}={\sum }_{\sigma }{b}_{k\sigma }^{\dagger }{a}_{k\sigma },{T}^{z}={\sum }_{k}({n}_{ka}-{n}_{kb})/2$ in particle-hole space. Precisely as in the *U* < 0 Anderson lattice^22^, one has a crossover to an incoherent exciton fluctuation dominated regime at ${k}_{B}T\simeq O(U^{\prime} )$, followed by a second crossover to a quasi-coherent exciton fluctuation dominated regime at a lower exciton-Kondo scale, ${T}_{K}^{ex}\simeq U^{\prime} {(\pi J\rho )}^{\mathrm{1/2}}{e}^{-\mathrm{1/(2}J\rho )}$, where *ρ* = *ρ*(*E*_*F*_) is the LDOS at *E*_*F*_ and $J\simeq {t}_{12}^{2}{\chi }_{12}\mathrm{(0)}$, with *χ*_12_(0) the excitonic susceptibility at *ω* = *E*_*F*_(=0): correlated FL behavior obtains below ${T}_{K}^{ex}$, thanks to an excitonic Kondo screening implicit in a situation where a tiny number of carriers are now coupled to fluctuating isospins *T*. It is precisely this resonant scattering that governs transport in the normal state in good accord with transport data as found above.

As *T* is lowered, intersite, residual interactions develop in full analogy with the way inter-site spin correlations, mediated by metallic carriers, arise in the Kondo lattice. They correspond to exchange coupling between isospins above, mediated by the tiny number of carriers, and are naturally viewed as an exciton fluctuation-exchange process. The residual interactions read^22^

3 $${H}_{eff}^{(2)}\simeq J\sum _{\langle i,j\rangle ,\sigma ,\sigma ^{\prime} }{a}_{i\sigma }^{\dagger }{b}_{j\sigma }{b}_{j\sigma ^{\prime} }^{\dagger }{a}_{i\sigma ^{\prime} }$$

which is also $J{\sum }_{\langle i,j\rangle ,\sigma ,\sigma ^{\prime} }[{n}_{ia\sigma }-{a}_{i\sigma }^{\dagger }{b}_{j,-\sigma }^{\dagger }{b}_{j\sigma }{a}_{i,-\sigma }]$. Absorbing the first term in the normal state Hamiltonian, ${H}_{res}^{\mathrm{(2)}}$ in momentum space is

$${H}_{res}^{(2)}=-J\sum _{kpq}({a}_{k\sigma }^{\dagger }{b}_{p,-\sigma }^{\dagger }{b}_{k-q,\sigma }{a}_{p+q,-\sigma }-{a}_{k\sigma }^{\dagger }{b}_{p,-\sigma }^{\dagger }{b}_{k-q,-\sigma }{a}_{p+q,\sigma })$$

Use of a usual BCS-like argument to derive SC from a static Hartree-Fock decoupling of ${H}_{res}^{\mathrm{(2)}}$ is possible^23^, but problematic. In particular, this is inappropriate in the case of *Bi* for the following reasons. The usual weak-coupling BCS approximation starts with the one-electron Green functions and constructs the “non-interacting” pair (particle-particle, p-p) susceptibility, composed from the lowest order bubble diagram involving the DFT one-electron propagators. Next, one uses this bare p-p susceptibility along with the *bare* attractive interaction (derived from integrating out appropriate bosons, *e*.*g*, phonons) to generate a ladder p-p vertex. The pole of this vertex function determines the SC transition temperature within BCS theory^3^. This procedure works well only as long as (*i*) the normal state above *T*_*c*_ is well-described by a weakly interacting Landau Fermi liquid, adiabatically connected to a free Fermi gas, and (*ii*) consequently, no quasi-bound state formation occurs in the *normal* state. Our results for the normal state in *Bi* above, showing good accord with spectral and transport responses, involve pre-formed, dynamically fluctuating excitons co-existing with a small number of carriers already in the normal state. Thus, the normal state in *Bi* does not satisfy condition (*ii*) above, making it mandatory to consider the strong coupling limit of the pairing problem beyond the BCS framework (see, *e*.*g*, Pietronero *et al*.^24^ for the non-adiabatic limit of the strong e-p coupling case).

As it stands, the effective interaction in a fluctuating excitonic “normal” state leads to possibility of both p-p and p-h condensation, and at intermediate-to-strong “excitonic Kondo” coupling, it is known that there is strong interference between both these channels at two-particle level. Then the associated logarithmic divergences appear in both channels, best illustrated within the classic parquet approach^25, 26^. We have adapted Abrikosov’s original parquet approach to our case (see SI for details). We proceed as follows: the bare vertex, ${{\rm{\Gamma }}}_{{\sigma }_{1}{\sigma }_{2}{\sigma }_{3}{\sigma }_{4}}^{\mathrm{(0)}}({p}_{1},{p}_{2},{p}_{3},{p}_{4})=J({\delta }_{{\sigma }_{1}{\sigma }_{3}}{\delta }_{{\sigma }_{2}{\sigma }_{4}}-{\delta }_{{\sigma }_{1}{\sigma }_{4}}{\delta }_{{\sigma }_{2}{\sigma }_{3}})$ will obtain drastic renormalization in the ‘excitonic Kondo’ regime. To this end, we exploit DMFT results: since the *a*-band spectral function is gapped, one can replace ${{\rm{G}}}_{aa}^{-1}(\omega )\simeq \omega +{\varepsilon }_{a}$ at low energy (since Im Σ_*aa*_(*ω*) = 0 at low energy), while the *b*-band propagator is approximated as ${G}_{bb}^{-1}(k,\omega )\simeq \omega -{z}_{b}{\varepsilon }_{k,b}$, with ${z}_{b}^{-1}=1-(d/d\omega )$Re ${\Sigma }_{bb}(\omega {)|}_{\omega ={E}_{F}}$. With these inputs, we find that the vertex is logarithmically enhanced, giving Γ(*ω*) = *J*[1 + *ρ*ln (*E*_*F*_/|*ω*| + *ε*_*a*_)]^−1^. The SC transition temperature is estimated from the divergence of the renormalized vertex, and we find *T*_*c*_ = *E*_*F*_.*e* ^−1/*Jρ*^. Using the *renormalized* ${E}_{F}\simeq {T}_{K}\simeq 100$ K (8.62 meV) from DMFT results below which correlated FL behavior sets in (instead of the *bare* ${E}_{F}\simeq 23$ meV (270 K)) in *Bi*, $\rho ({E}_{F})\simeq 0.1$ eV^−1^ from normal state DMFT results, and the coupling $J\simeq O\mathrm{(1)}$ (we are *not* in the regime *t*_11,22,12_ ≪ *U*′ for *Bi*), we estimate the SC ${T}_{c}\simeq O\mathrm{(1)}$ mK, quite close to the experimental finding of ${T}_{c}\simeq 0.5$ mK. Given the approximations made above, this is very reasonable. Our estimate is a huge enhancement compared to the ${T}_{c}^{BCS}\simeq O\mathrm{(100)}$ nK found using the naive BCS formula^3^, and reflects the strong coupling nature of SC, where non-adiabatic effects enhance *T*_*c*_ via huge enhancement of the vertex. Effects akin to the above have been discussed in the non-adiabatic limit of strong electron-phonon coupling^24, 27–29^ and, in *Bi*, the fact that $g/{E}_{F}\simeq O\mathrm{(0.5)}$ will also imply such additional enhancement arising from electron-phonon coupling. In the adiabatic or anti-adiabatic limit, it is possible to subsume this latter effect into a renormalization of the bare vertex *J*. However, when $g/{E}_{F}\simeq O\mathrm{(1)}$, one cannot “integrate out” the phonons. One must include e-p couplig explicitly in such a case and, in fact, our DMFT results do include the renormalization caused by e-p coupling as well. Such strong coupling multi-band SC will also generally give an enhanced (*dH*_*c*2_/*dT*) in *Bi* ^3^, as in other documented near semi-metallic superconductors^30^, relative to the BCS prediction.

Finally, the form of the effective interaction also shows that the excitonic insulator (EI) phase is a subleading instability in *Bi*. It is of interest to inquire whether evidence for such a “competing order” could obtain in *Bi* by modification of its electronic structure, *e*.*g*, by pressure^1^ or an external magnetic field. Both reduce the tiny carrier density further, jacking up the effective *U*/*t* ratio and inducing tendency to localization. Actually, pressure does induce a metal-insulator-like transition in Bismuth^1^. Within our strong coupling analysis, the competing order in the resultant semiconducting phase would be characterized by an order parameter ${{\rm{\Delta }}}_{exc}={\sum }_{k,\sigma }\langle \,{f}_{12}(k){c}_{1k\sigma }^{\dagger }{c}_{2k\sigma }\rangle$ with ${f}_{12}(k)=2\sqrt{3}$sin$(\sqrt{3}{k}_{x}a/2)$sin (*k*_*y*_ *a*/2). This excitonic order parameter does not break inversion symmetry (*k* → −*k*) but, remarkably, is associated with an electronic *nematic* order^22^. Very recent work^31^ finds a nematic electronic state with a tiny gap *O*(500)*μ*eV on the surface of *Bi* under high magnetic fields. Whether a Rashba-SOC modified electronic structure as above can induce an excitonic instability as proposed here, and whether such a state can lead to a reconstructed electronic structure having the observed anisotropy of Landau level wave-functions, is an enticing open issue. Thus, future studies under pressure can confirm or refute our prediction which, at this time seems to have limited confirmation^31^. Investigation into these aspects is left for future work.

## Electronic supplementary material

Dramatically Enhanced Superconductivity in Elemental Bismuth from Excitonic Fluctuation Exchange Supplementary Information

## References

[1] Armitage NP (2010). Infrared conductivity of elemental bismuth under pressure: Evidence for an avoided Lifshitz-type semimetal- semiconductor transition. Physical Review Letters.

[2] Behnia K, Balicas L, Kopelevich Y (2007). Signatures of electron fractionalization in ultraquantum bismuth. Science.

[3] Prakash O, Kumar A, Thamizhavel A, Ramakrishnan S (2017). Evidence for bulk superconductivity in pure bismuth single crystals at ambient pressure. Science.

[4] Barrett CS (1960). The Structure of Bismuth at Low Temperature. Australian Journ. Phys..

[5] Gonze X, Michenaud J-P, Vigneron J-P (1990). First-principles study of As, Sb, and Bi electronic properties. Physical Review B.

[6] Xu JH, Wang EG, Ting CS, Su WP (1993). Tight-binding theory of the electronic structures for rhombohedral semimetals. Physical Review B.

[7] Kukkonen CA, Sohn KF (1977). The low-temperature electrical resistivity of bismuth. Journal of Physics F: Metal Physics.

[8] Chudzinski P, Giamarchi T (2011). Collective excitations and low-temperature transport properties of bismuth. Physical Review B.

[9] Tediosi R, Armitage NP, Giannini E, Van Der Marel D (2007). Charge carrier interaction with a purely electronic collective mode: Plasmarons and the infrared response of elemental bismuth. Physical Review Letters.

[10] Georges A, Kotliar G, Krauth W, Rozenberg MJ (1996). Dynamical mean-field theory of strongly correlated fermion systems and the limit of infinite dimensions. Review of Modern Physics.

[11] Cohen ML (1964). Superconductivity in many-valley semiconductors and in semimetals. Physical Review.

[12] Taraphder A, Koley S, Vidhyadhiraja NS, Laad MS (2011). Preformed Excitonic Liquid Route to a Charge Density Wave in 2H-TaSe_2_. Physical Review Letters.

[13] Koley S, Laad MS, Vidhyadhiraja NS, Taraphder A (2014). Preformed excitons, orbital selectivity, and charge density wave order in 1T-TiSe_2_. Physical Review B.

[14] Koley S, Mohanta N, Taraphder A (2015). The unusual normal state and charge-density-wave order in 2H-NbSe_2_. Journal of Physics: Condensed Matter.

[15] Dasari N (2016). A multi-orbital iterated perturbation theory for model Hamiltonians and real material-specific calculations of correlated systems. Eur. Phys. J. B.

[16] Bauer B (2011). “The ALPS project release 2.0: open source software for strongly correlated systems”. Journal of Statistical Mechanics: Theory and Experiment.

[17] Keller M, Metzner W, Schollwock U (2001). Dynamical mean-field theory for pairing and spin gap in the attractive hubbard model. Physical Review Letters.

[18] Kancharla SS, Okamoto S (2007). Band insulator to Mott insulator transition in a bilayer Hubbard model. Physical Review B.

[19] Das SD (2015). Quantum criticality in the 122 iron pnictide superconductors emerging from orbital-selective Mottness. Physical Review B.

[20] de’ Medici L, Hassan SR, Capone M, Dai X (2009). Orbital-selective Mott transition out of band degeneracy lifting. Physical Review Letters.

[21] Tomczak JM, Biermann S (2009). Optical properties of correlated materials: generalized Peierls approach and its application to VO_2_. Physical Review B.

[22] Taraphder A, Coleman P (1991). Heavy-fermion behavior in a negative-U Anderson model. Physical Review Letters.

[23] Koley S (2017). Pressure Driven Phase Transition in 1T-TiSe_2_, a MOIPT+DMFT Study. Solid State Communications.

[24] Pietronero L, Strassler S (1992). Theory of nonadiabatic superconductivity. Europhysics Letters.

[25] Bychkov YA, Gor’kov LP, Dzyaloshinskii IE (1995). Possibility of superconductivity type phenomena in a one-dimensional system. World Scientific Series in 20th Century Physics.

[26] Svozil K (1988). Heavy Fermion Superconductivity via Kondo Type Pairing. Physica status solidi (b).

[27] Durajski AP (2016). Quantitative analysis of nonadiabatic effects in dense H3S and PH3 superconductors. Scientific Reports.

[28] Grimaldi C, Pietronero L, Scattoni M (1999). The physical origin of the electron-phonon vertex correction. European Physical Journal B-Condensed Matter and Complex Systems.

[29] Miller P, Freericks JK, Nicol EJ (1998). Possible experimentally observable effects of vertex corrections in superconductors. Physical Review B.

[30] Singh DJ (2015). Multiband Semimetallic Electronic Structure of Superconducting Ta_2_PdSe_5_. PloS one.

[31] Feldman BE (2016). Observation of a nematic quantum Hall liquid on the surface of bismuth. Science.

