# Supplementary material for: Dramatically Enhanced Superconductivity in Elemental Bismuth from Excitonic Fluctuation Exchange
Source: Sci Rep. 2017 Sep 8;7:10993. doi: 10.1038/s41598-017-11269-y (PMC5591209; doi:10.1038/s41598-017-11269-y)
Supplement: Supplementary file 1 — Dramatically Enhanced Superconductivity in Elemental Bismuth from Excitonic Fluctuation Exchange Supplementary Information [file 41598_2017_11269_MOESM1_ESM.pdf]

Dramatically Enhanced Superconductivity in Elemental Bismuth from Excitonic Fluctuation Exchange
Supplementary Information

S. Koley[3,*] M. S. Laad[1,†] and A. Taraphder[2‡]

[1]Instt. Math. Sciences Taramani, Chennai, 600113 and Homi Bhabha National Institute, India
[2]Department of Physics and Centre for Theoretical Studies,
Indian Institute of Technology, Kharagpur, 721302 India and
[3] Department of Physics, St. Anthony's College, Shillong, Meghalaya, 793001 India

A. Electron-hole and electron phonon coupling within Two-Band DMFT(IPT)

Our starting point for the DMFT including electron-electron, electron-hole and electron phonon couplings in Bismuth on a single footing starts with

(i) the tight-binding band-structural input as described in the main text, with the tight-binding bands adapted from earlier work[8]. We have included the two bands closest to the Fermi energy (E_F), since only these will be important at low temperature, T, of relevance to Bi. This constitutes an adequate input for the one-electron band structure, and reproduces the Fermi surface of Bi very well.

(ii) Next, we turn to the effects of $e-e, e-h$ and electron-phonon interactions in Bi. Since it is known (see main text) that the interactions between valence- and conduction band carriers in Bi is predominantly of short-range, excitonic character, a local approximation in the spirit of dynamical mean-field theory (DMFT) is adequate to capture these correlation effects. The dynamical correlations associated with these local interactions are embodied in a dynamical self-energy, which contains information about the finite energy and T-dependence of the excitations in the system. The spectrum of these elementary excitations controls the finite temperature and dynamical responses of an interacting many-body system.

(iii) We have employed the multi-orbital iterated perturbation theory (MO-IPT) as an "impurity solver" for the DMFT equations. The detailed procedure has already been worked out in detail, and applied to a wide-ranging class of real, strongly correlated systems, and so we present an adaptation of the scheme relevant for Bi in what follows.

Computation of the self-energy for multi-band Hubbard models using the iterated perturbation theory (IPT) has already been done by many authors[1,2], so we refer to earlier work in this context. Moreover, multi-band IPT has recently been benchmarked against more numerically exact continuous-time quantum Monte Carlo (CTQMC) solver for DMFT. It has been found that excellent accord with IPT and CTQMC solvers obtain in physical situations with finite crystal-field splitting between one-electron (DFT) bands and/or finite Hund coupling. In Bismuth, the role of this "crystal field" splitting (which generically translates into removal of band degeneracy) is played by the energy difference between the valence and conduction bands. Thus, we use the multi-orbital IPT solver to account for electronic correlation effects and, in particular, to rationalize the effect of strong scattering of fermions on dynamical, preformed excitonic fluctuations. To find electron-phonon self-energy we follow the procedure used by Ciuchi et al.[3] (using Einstein phonons) into our multi-orbital DMFT formulation. The intra- and inter-orbital Coulomb correlations are considered upto second order. Since both Coulomb and electron-phonon interaction are local, their combined effect can be treated simultaneously within self consistent DMFT. The electronic self energy is modified here due to electron-phonon coupling with the term

$$g^2\sum_{i\omega_n}G_0(p)D_0(\omega)=g^2[\frac{N_q+n_f(\zeta_p)}{ip_n+\omega_q-\zeta_p}+\frac{N_q+1-n_f(\zeta_p)}{ip_n-\omega_q-\zeta_p}]$$

Where $N_q=\frac{1}{e^{\beta\omega_q}-1}$, g is electron-phonon coupling .
Now the full Hamiltonian, with exciton-phonon coupling is

$$H=\sum_{k,a,b,\sigma}(t_k^{ab}+\epsilon_a\delta_{ab})c^{\dagger}_{ka\sigma}c_{kb\sigma}+U\sum_{i,\mu=a,b}n_{i\mu\sigma}n_{i\mu-\sigma}$$

$$+U_{ab}\sum_i n_{ia}n_{ib}+g\sum_i(c^{\dagger}_{a\sigma}c_{b\sigma}+h.c.)(A_i^{\dagger}+A_i)$$

$$-V\sum_i c^{\dagger}_{b\sigma}c_{b\sigma}(1-c^{\dagger}_{a\sigma}c_{a\sigma})+\omega_0\sum_i A_i^{\dagger}A_i.$$

where a and b are band indices, U is intra-orbital correlation and U_{ab} is the inter-orbital correlation term along with inter-band hopping t_k^{ab}. The electron lattice part of the Hamiltonian is $g\sum_i(c^{\dagger}_{a\sigma}c_{b\sigma}+h.c.)(A_i^{\dagger}+A_i)$ (coupling of A_{1g} phonons to the inter-band excitons. We initiate ansatz for the self energy $\Sigma_{int}(\omega)=Un+A\Sigma_0^{(2)}(\omega)$ where $\Sigma_0^{(2)}(\omega)$ is the second order contribution of electron-electron and exciton-phonon coupling. The IPT scheme is to find lattice Green's function (G_{fa}) from this full self energy, and then bare Green's function is found via the Dyson equation: $G_{0a}^{-1}=G_{fa}^{-1}+\Sigma_a$. Using this G_{0a} into the IPT, we obtain a new estimate of $\Sigma_{0a}^{(2)}$ and this procedure is iterated till it reaches self consistency.

Following usual definition, A_{ab} is established from the condition that it reproduce the leading behavior of the (of the exact atomic limit) self-energy at high frequency. The leading behavior for large ω can be obtained by expanding the Green function in a continuous fraction[4]: $G_{fa}(k,\omega) = 1/(\omega - \epsilon_{fa} - M_{1a} - \frac{M_{2a}-M_{1a}^2}{\omega+...})$ where, M_i denotes the ith order moment of the density of states(computed by evaluating a commutator[5], and for the model above, $M_{2a} - M_{1a}^2 = U_{ab}^2(n_{fa}(1-2n_{fa}) + <n_{fa}n_{fb}>) + g^2(n_{fa+} + n_{fa-}))$. Here, n_{fa} is the number density calculated from the full Green's Function, $n_{fa+}= g^2\sum_{\omega}(G_f(\omega+\omega_q)(N_q + n_f(\omega))$ and $n_{fa-}= g^2\sum_{\omega}(G_f(\omega-\omega_q)(N_q + n_f(-\omega))$. From the large frequency limit of (1), $\Sigma_{0a}^2(\omega) = U_{ab}^2 n_{0a}(1-n_{0a}) + g^2(n_{0a-} + n_{0a+})$. Here, n_{0a} is a fictitious number density of the 'bare' Green's function. Explicitly, $n_{0a+}= g^2\sum_{\omega}(G_0(\omega+\omega_q)(N_q + n_f(\omega))$ and $n_{0a-}= g^2\sum_{\omega}(G_0(\omega-\omega_q)(N_q + n_f(-\omega))$. Comparing with the exact high-frequency limit, we thus have $A_{ab} = \frac{U_{ab}^2(n_{fa}(1-2n_{fa})+<n_{fa}n_{fb}>)+g^2(n_{fa+}+n_{fa-})}{U_{ab}^2 n_{0a}(1-n_{0a})+g^2(n_{0a-}+n_{0a+})}$.

B. Parquet approach

Here, we adapt the parquet approach of Bychkov *et al.*[7] to our model. In the parquet approach[7], graphs corresponding to p-h and p-p vertices which cannot be cut into two separate pieces by cutting two (a, the "heavy" band, or b, the metallic band in Bi) propagator lines are neglected. However, the leading logarithmic corrections arising from Kondo screening are retained. At Nth order, the magnitude of each diagram for $\Gamma$is x^N, where $x = J\rho\int G_{aa}G_{bb}d\omega d\epsilon \simeq J\rho \ln(E_F/\omega + \epsilon_a) \simeq O(1)$. The full vertex in the parquet approximation is $\Gamma = \Gamma_0 + \Lambda_{pp} + \Lambda_{ph}$, with Γ_0 the bare vertex, Λ_{pp} the p-p "brick" which can be cut by two parallel a, b propagator lines, and Λ_{ph} the corresponding brick in the p-h channel. The parquet series is analyzed by requiring (i) no energy transfer to and from the internal (gapped) a-band (gapped in case of Bi, see DMFT results) states; thus, one can set $\omega_2 = \omega_3 = 0$ and $\omega_1 = \omega_4 = \omega$, ($ii$) all momenta $p_i, i = 1,2,3,4$ are set equal to p_F. (iii) using $\Gamma(\omega,0,0,\omega) = \Gamma(\omega)$, and choosing an *internal* two-line state such that the energy ω' of the G_{aa} line is minimum. Since there is a *full* vertex part $\Gamma(\omega')$ to the left and right of this part, one finds[7],

$$\Gamma^P_{\sigma_i}(\omega) = \rho\int_{|\omega|}^{E_F}\frac{\Gamma_{\sigma_1\sigma_4\mu\nu}(\omega')\Gamma_{\mu\nu\sigma_2\sigma_3}(\omega')}{\omega'}d\omega' \quad (1)$$

and

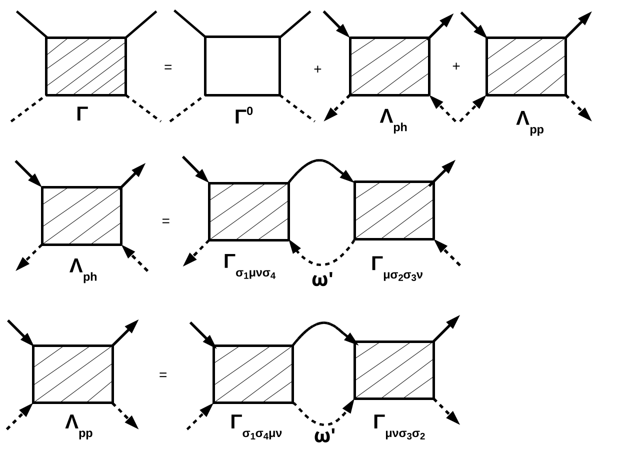

FIG. 1. The diagrammatic representation of the parquet approximation for the full vertex function (upper figure) and the blocks in the particle-hole (ph, center figure) and particle-particle (pp, lower figure) channels (see text for details).

$$\Gamma_{\sigma_i}(\omega) = \rho\int_{|\omega|}^{E_F}\frac{\Gamma_{\sigma_1\nu\mu\sigma_4}(\omega')\Gamma_{\mu\sigma_2\sigma_3\nu}(\omega')}{\omega'}d\omega' \quad (2)$$

Using the spin-separable structure of Γ, one obtains $\Gamma(x) = J - J\rho\int_0^x \Gamma^2(x')dx'$, with $x = \ln(E_F/|\omega| + \epsilon_a)$. This has the solution $\Gamma(\omega) = J[1 + \rho\ln(E_F/|\omega| + \epsilon_a)]^{-1}$, showing up the logarithmic enhancement of the two-particle vertex. Hence, with $J > 0$ (notice that this co-efficient of $H_{res}^{(2)}$ is *negative* as above), $\Gamma(\omega)$ has a pole when $\Gamma^{-1}(\omega_c = T_c) = 0$, signalling an instability to the superconducting state when $T_c \simeq E_F.e^{-1/J\rho}$. Thus, this enhancement of T_c relative to the simple weak-coupling BCS estimate is a consequence of the logarithmic enhancement o the vertex function. Using the *renormalized* $E_F \simeq T_K \simeq 100$ K from DMFT results below which correlated FL behavior sets in (instead of the *bare* $E_F \simeq 23$ meV in Bi), $\rho(E_F \simeq 0.1\text{eV}^{-1}$, and the coupling $J \simeq O(1)$) (we are *not* in the regime $t_{11,22,12} << U'$ for Bi), we estimate the SC $T_c \simeq O(1)$ mK, quite close to the experimental finding of $T_c \simeq 0.5$ mK.

The above parquet analysis also suggests that T_c could be enhanced further if it would be possible to move the a-band states (ϵ_a) closer to the Fermi energy by appropriate perturbations.

C. Tight Binding Matrix elements

In rhombohedral A7 structure bismuth crystal, there are two atoms in the primitive unit cell. In the semimetal bismuth we consider only one set of s, p_x, p_y, and p_z orbitals at each atom. So the Hamiltonian matrix will contain all possible nearest neighbour, next nearest neighbour and third nearest interactions between the tight-binding basis functions centered on each atom of the unit

cell and will form 8×8 matrix, which can be calculated from the Slater and Koster approach,

$$H_{\alpha\alpha'}(k) = \sum_i e^{ik.r_i} < 0, \alpha|H|i, \alpha' > \quad (3)$$

Now we need the atomic matrix elements $< 0, \alpha|H|i, \alpha' >$. Following the Slater-Koster approach and adjusting chemical potential the tight binding bands can be fit to the extant LDA calculation. The calculated matrix elements are

$\epsilon 11 = E_s + V''_{ss\sigma}(2cos(y) + 4cos(y/2)cos(\sqrt{3}x/2))$
$\epsilon 12 = i2\sqrt{3}V''_{sp\sigma}cos(y/2)sin(\sqrt{3}x/2)$
$\epsilon 13 = i2V''_{sp\sigma}(sin(y) + sin(y/2)cos(\sqrt{3}x/2))$
$\epsilon 13 = 0$
$\epsilon 15 = V_{ss\sigma}(e^{i\zeta_1} + 2cos(y/2)e^{-i\eta_1}) + V'_{ss\sigma}(e^{-i\zeta_2} + 2cos(y/2)e^{i\eta_2})$
$\epsilon 16 = V_{sp\sigma}A_1(e^{i\zeta_1} - cos(y/2)e^{-i\eta_1}) - V' sp\sigma A_2(e^{-i\zeta_2} - cos(y/2)e^{i\eta_2})$
$\epsilon 17 = i\sqrt{3}V_{sp\sigma}A_1 sin(y/2)e^(- i\eta_1) + i\sqrt{3}V'_{sp\sigma}A_2 sin(y/2)e^(i\eta_2)$
$\epsilon 18 = -\sqrt{3}V_{sp\sigma}c_1A_1(e^{i\zeta_1} + 2cos(y/2)e^{-i\eta_1}) + \sqrt{3}V' sp\sigma(e^{-i\zeta_2} + 2cos(y/2)e^{i\eta_2})$
$\epsilon 22 = E_p + 3V''_{pp\sigma}cos(\sqrt{3}x/2)cos(y/2) + V''_{pp\pi}(2cos(y) + cos(y/2)cos(\sqrt{3}x/2))$
$\epsilon 23 = -\sqrt{3}(V''_{pp\sigma} - V''_{pp\pi})sin(\sqrt{3}x/2)sin(y/2)$
$\epsilon 24 = 0$
$\epsilon 25 = -\epsilon 16$
$\epsilon 26 = \frac{1}{2}V_{pp\sigma}A_1^2(2e^{i\zeta_1)} + cos(y/2)e^{-i\eta_1}) + 3V_{pp\pi}A_1^2c_1^2(e^{i\zeta_1)} + 2(1 + \frac{1}{4c_1^2})cos(y/2)e^{-i\eta_1}) + \frac{1}{2}V'_{pp\sigma}A_2^2(2e^{-i\zeta_2)} + cos(y/2)e^{i\eta_2}) + 3V'_{pp\pi}A_2^2c_2^2(e^{-i\zeta_2)} + 2(1 + \frac{1}{4c_2^2})cos(y/2)e^{i\eta_2})$
$\epsilon 27 = -\frac{\sqrt{3}}{2}(V_{pp\sigma} - V_{pp\pi})A_1^2 sin(y/2)e^{-i\eta_1} + \frac{\sqrt{3}}{2}(V'_{pp\sigma} - V'_{pp\pi})A_2^2 sin(y/2)e^{i\eta_2}$
$\epsilon 28 = -\sqrt{3}(V_{pp\sigma} - V_{pp\pi})A_1^2c_1(e^{i\zeta_1} - cos(y/2)e^{-i\eta_1}) - \sqrt{3}(V'_{pp\sigma} - V'_{pp\pi})A_2^2c_2(e^{-i\zeta_2} - cos(y/2)e^{i\eta_2})$
$\epsilon 33 = E_p + V''_{pp\sigma}(2cos(y) + cos(y/2)cos(\sqrt{3}x/2)) + 3V''_{pp\pi}cos(y/2)cos(\sqrt{3}x/2)$
$\epsilon 34 = 0$
$\epsilon 35 = -\epsilon 17$
$\epsilon 36 = \epsilon 27$
$\epsilon 37 = \frac{3}{2}V_{pp\sigma}A_1^2cos(y/2)e^{-i\eta_1} + V_{pp\pi}(6A_1^2(c_1^2 + \frac{1}{4})cos(y/2)e^{-i\eta_1} + e^{i\zeta_1}) + \frac{3}{2}V_{pp\sigma}A_1^2cos(y/2)e^{-i\eta_1} + V'_{pp\pi}(6A_2^2(c_2^2 + \frac{1}{4})cos(y/2)e^{i\eta_2} + e^{-i\zeta_2})$
$\epsilon 38 = -i3(V_{pp\sigma} - V_{pp\pi})A_1^2c_1 sin(y/2)e^{-\eta_1} - i3(V_{pp\sigma} - V_{pp\pi})A_2^2c_2 sin(y/2)e^{\eta_2}$
$\epsilon 44 = E_p + V''_{pp\pi}(2cos(y) + cos(y/2)cos(\sqrt{3}x/2))$
$\epsilon 45 = -\epsilon 18$
$\epsilon 46 = \epsilon 26$
$\epsilon 47 = \epsilon 38$
$\epsilon 48 = 3V_{pp\sigma}A_1^2c_1^2(e^{i\zeta_1)} + 2cos(y/2)e^{-i\eta_1}) + V_{pp\pi}A_1^2(e^{i\zeta_1)} + 2cos(y/2)e^{-i\eta_1}) + 3V'_{pp\sigma}A_2^2c_2^2(e^{-i\zeta_2)} + 2cos(y/2)e^{i\eta_2}) + V'_{pp\pi}A_1^2(e^{-i\zeta_2)} + 2cos(y/2)e^{\eta_2})$
$\epsilon 55 = \epsilon 11$
$\epsilon 56 = \epsilon 12$
$\epsilon 57 = \epsilon 13$
$\epsilon 58 = 0$
$\epsilon 66 = \epsilon 22$
$\epsilon 67 = \epsilon 23$
$\epsilon 68 = 0$
$\epsilon 77 = \epsilon 33$
$\epsilon 78 = 0$
$\epsilon 88 = \epsilon 44$

where $\zeta_{1,2} = x/\sqrt{3} - c_{1,2}z, \eta_{1,2} = x/2\sqrt{3} + c_{1,2}z, A_{1,2} = \frac{1}{1+3c_{1,2}^2}$, c_1=1.5896, c_2=2.3426 and other constants are given in tableI.

* sudiptakoley20@gmail.com
† mslaad@imsc.res.in
‡ arghya@phy.iitkgp.ernet.in

[1] Laad, M.S., Craco, L. and Müller-Hartmann, E., Orbital Switching and the First-Order Insulator-Metal Transition in Paramagnetic V_2O_3. *Phys. Rev. Lett.*, **91**, 156402 (2003).
[2] Dasari, N. et al. A multi-orbital iterated perturbation theory for model Hamiltonians and real material-specific calculations of correlated systems. *Eur. Phys. J. B*, **89**, 202 (2016).
[3] Ciuchi, S., et al., Superconductivity and Density Waves in High Dimensions. *Europhys. Lett.*, **24** 575 (1993).
[4] Gordon, R.G., Error Bounds in Equilibrium Statistical Mechanics . *J. Math. Phys. (N.Y.)*, **9**, 655 (1968).
[5] Nolting, W. and Borgie, W., Band magnetism in the Hubbard model. *Phys. Rev. B*, **39**, 6962 (1989).
[6] Gunnarsson, O., and Rösch, O., Interplay between electron phonon and Coulomb interactions in cuprates. *J. Phys. Condens. Matter*, **20**, 043201 (2008).
[7] Bychkov, Yu., Gorkov, L. and Dzyaloshinskii, I., Possibility of superconductivity type phenomena in a one-dimensional system. *J. Exptl. Theor. Phys. (U.S.S.R)* **50**, 738 (1966); Svozil, K., Heavy Fermion Superconductivity via Kondo-Type Pairing. *Physica Status Solidi*, **147**, 635 (1988).
[8] Xu, J.H., Wang, E.G., Ting, C.S. and Su, W.P. Tight-binding theory of the electronic structures for rhombohedral semimetals. *Physical Review B*, **48**, 17271 (1993).

E_s	E_p	$V_{ss\sigma}$	$V_{sp\sigma}$	$V_{pp\pi}$	$V_{pp\sigma}$	$V'_{ss\sigma}$	$V'_{sp\sigma}$	$V'_{pp\pi}$	$V'_{pp\sigma}$	$V''_{pp\pi}$	$V''_{pp\sigma}$	$V''_{sp\sigma}$
-9.643	-0.263	-0.703	1.3	-0.679	2.271	-0.275	0.108	-0.337	1.42	0.065	0.004	0.303

TABLE I. Tight Binding Matrix elements (in eV) for Bismuth[8]
